# Bibliographical Notices

**Published:** 1842-07-01

**Authors:** 


### Periscope;

OR,

### CIRCUMSPECTIVE REVIEW.

" Ore trahit quodcunque potest, atque addit acervo."

### Notices of some New Works.

Observations on the Admission of Medical Pupils to the Wards of Bethlem Hospital, for the purpose of Studying Mental Diseases. By *John Webster*, M.D.

We believe that there are very few modern physiologists who now consider insanity as a disease of the *mind alone*, but as a disorder of some material structure, more especially of the mind's instrument—the brain. Sensation, volition, and reflection, are just as much functions of the brain and nerves, as secretion of bile is the function of the liver—or circulation of blood the function of the heart and arteries. It is very true that the primary *cause* of insanity may not always be in the brain. It may be in the liver, the stomach, or other parts; but the brain must be disordered, either primarily or sympathetically, before insanity can manifest itself. The functions of the brain, like those of other organs or parts, may be *disordered*, long before the microscope or scalpel can detect changes of *structure*—which, after all, are consequences, not causes. These are truths which we believe are almost universally admitted, and yet they have not led to the legitimate conclusion that, insanity being a disease or disorder of the mind's instrument—the brain, wherever located may be the original cause—so the complaint ought to be taught in lectures and studied in hospitals like any other corporeal malady.

Dr. Webster has, therefore, done the state, as well as the profession, a service by bringing this subject before the public, in the sensible pamphlet under review. The following passage from Sir W. Ellis, is so much *ad rem* that we shall here quote it.

" It is perfectly inconsistent with common sense to suppose, that a man shall intuitively know how to treat insanity. We have seen, that although in the greater number of cases it is attended with the same general result, yet it assumes most varied forms, and great care and discrimination are required in the treatment; indeed, it is universally acknowledged to be a most difficult and mysterious disease, and yet it is almost the only one on which the medical student receives no particular instruction. In his attendance on the hospitals, he will, in all probability, have met with almost every other variety of disease which afflicts human nature; at all events, his lectures will have supplied him with some information as to their treatment; but I believe that my friend and colleague, Dr. (now Sir Alexander) Morison, of Cavendish Square, is the only lecturer in London expressly on insanity.—Indeed, excepting as being incidently touched upon in the lectures on forensic medicine, it appears almost entirely neglected in the course of a medical education; and, as the subject does not form a branch of examination, the pupils naturally employ their time in those studies which will be directly available, and assist them in the obtaining their medical certificates; the result is, that professional men, in other respects well educated, commence practice almost in a state of total ignorance on the subject. This is an evil from which every individual, whatever be his rank and fortune, is liable to suffer in his own person, and in that of his friends; and a man of ingenious mind can hardly be placed under more painful circumstances, than to find the father or mother of a family in a state of insanity, entrusted to his care, and to feel conscious that upon him depends the restoration of the patient to reason and happiness, whilst his want of acquaintance with the disease renders him unfit for the task, and he knows not where to apply for advice. This is by no means an imaginary evil, it is one of frequent occurrence, and numerous are the instances where amiable and valuable members of society are consigned for life, to a perpetual banishment from their friends in the gloom of a mad-house, solely from ignorance on the part of the medical adviser. This ought to be remedied."

But it will be said, "Oh! insanity is a disease of the mind, and must be cured by moral means." We deny the fact, or rather the fiction. In the first place, insanity is *not* a disease of the mind, but of its organ. In the second place, although moral means are, perhaps, more important auxiliaries in the treatment of a complaint affecting the organ of thought than of one affecting an organ of circulation, secretion, or absorption, yet still they are only auxiliaries, and will not succeed in one case out of ten, without physical remedies. And how are the causes and phenomena of the disease, or the adaptation of remedies to be learnt but by the same process as in other corporeal ailments—namely, by precept and example?

Till the curricula of the medical corporations include lectures and clinical instruction in this branch of medical science, it is not to be expected that students will search for information beyond the sphere of their ordinary teachers, or that lunatic hospitals or asylums will open their doors to the clinical inquirer. Since Dr. Webster wrote this brochure, we are happy to see that a course of lectures on insanity has been instituted by Dr. Conolly, and we sincerely hope that this auspicious beginning will be followed by the opening of wards for clinical observation at the great institutions, metropolitan and provincial, dedicated to the use of insane people, of both sexes.

Dr. Webster properly observes that Bethlem Hospital is peculiarly well calculated for affording excellent clinical instruction. The wards contain generally from 310 to 340 patients—and the wards are designed for the reception of cases that are probably curable—not a receptacle for those who are beyond all hope of recovery. The objection made by governors, that the sight of pupils going round with physicians migh excite, and thus injure the insane, is quite futile. No such effect is produced in general hospitals, even where patients are suffering from violent or acute diseases—or undergoing formidable operations. We quite concur with Dr. Webster in the following remarks.

"So far from considering, that the judicious admission of pupils, and of young men who are about completing their preliminary studies, to the wards of Bethlem Hospital, would prove injurious to the inmates, I believe, if properly regulated, such permission would even sometimes act advantageously. This opinion is founded upon the supposition, that the regular visits of the physicians, although accompanied by pupils, would in some of the patients tend rather to distract the attention from their false reasoning, whilst in others it would appear as if bringing them in contact with the external world, and so produce a more favourable impression upon their disordered imaginations. Besides the appearance thus given to the establishment, of being somewhat like an ordinary hospital, for the restoration to health of its inmates, instead of a common mad house, in which all contact with the world outside its walls is generally cut off, might have a favourable impression; especially as it is a well known observation, that the fear of being placed in a lunatic asylum, often exerts an injurious influence upon the minds even of the insane; to say nothing of the very disagreeable associations, which it produces upon friends and relatives."

Dr. Webster concludes his pamphlet with some judicious remarks on the management of the insane at this period as compared with that pursued at the beginning even of the present century. Whilst he properly condemns chains, dark cells, handcuffs, &c. he candidly acknowledges that—

" When an insane person is either dangerous to others, or likely to inflict injury upon himself, restraint may, in that case, become necessary."

This is all we have ever contended for, in the way of restraint. We return our best thanks to Dr. Webster for his sensible and well-timed letter, a second edition of which is already published.

---

### First and Second Reports of the Medical Missionary Society in China. Macao, 1841.

A few years ago an hospital was opened at Canton, and occasionally at Macao, where, up to the date of report, more than six thousand sick received relief. Ophthalmic diseases are very frequent in China, and these formed a large proportion of the cases treated.

" A large number of them have been restored from partial or total blindness to all the blessings of good and useful sight. The almost uniform success of the medical and surgical treatment at the institutions of the Society, the growing confidence of the Chinese, which is the natural result of this, their grateful sense of the benefits conferred upon them through the skill and philanthropy of foreigners,—are so many powerful encouragements to perseverance in the pursuit of the noble objects for which we are united. And so persevering, we may look forward with confidence to the time, when, having afforded to the intelligent youth of China a good medical education, we shall no longer confine our efforts to the small circle within which our residence is now circumscribed, but may be enabled first to send forth our practitioners, and ultimately perhaps to follow them ourselves, through the length and breadth of the empire."

The chief medical officers of these establishments are Dr. Parker, Mr. Lockhart, and Mr. Hobson, to whom great praise is due for their zeal, talents, and humanity. Their exertions will do more to harmonize the two mighty empires of Britain and China than all our naval and military expeditions, however brilliant or victorious. The following passage from a report of Dr. Parker's is interesting.

" Often has the sincerest gratitude been felt towards the benevolent members of this Society, who have procured such an asylum for the afflicted Chinese, and to the respected President whose judgment first selected the premises, when,—walking through its capacious and numerous apartments,—I have witnessed the comfortable accommodation afforded to the inmates, to many of whom it seemed almost a palace, in comparison with the narrow cells they call their homes. The building is capable of accommodating two hundred patients. It has nineteen spacious rooms on the second story, well ventilated; and as many corresponding ones on the ground floor; a garden, and extensive compound, with three wells of water—in the rear; and a yard in front. The building is of brick, strongly built, and the whole of the ground (say a third of an acre) belonging to it, is surrounded by a substantial wall. It is in a healthy locality, overlooking the waters of the inner harbour, and having good access both by land and water."

Notwithstanding the prejudices, ignorance, and hatred to foreigners which the Chinese exhibit, it appears that they pay the greatest respect to English medical men, and place in them the most implicit confidence. Crowds of Chinese, of both sexes, afflicted with all kinds of disorders, are seen soliciting aid in the attitude of humility and respect, listening to advice.

"To behold a female, unaccompanied perhaps by a single friend or relative, brought in and tied hand and foot to the operator's table, and there submit to a most painful operation, without uttering a sigh or a groan, teaches us, in terms that can neither be misunderstood nor prevaricated, that a Chinese, upon proper grounds, is able to exercise the most unbounded confidence in the wisdom and goodness of the stranger."

### CHUSAN.

This island, now so famous, having come under the range of British influence, it was deemed desirable to establish an hospital there, which was completed in February 1841, under the direction of Mr. Lockhart. No less than 3502 patients were attended to—sometimes as many as 200 old and new cases presenting themselves in one day! At first, indeed, the inhabitants could not comprehend the object of the hospital; but attention to some of the sick met in the streets, soon enlightened their understandings, and they flocked in numbers to the establishment for medical and surgical relief. Several were attended also at their own homes, by which a nearer insight into the domestic habits and economy of this curious people was obtained than could have been done under any other circumstances. Patients, in a little time, came from towns and villages far remote from Chusan.

In the south-west monsoon the weather was very hot, and sometimes oppressive—the thermometer standing at 90 in the shade by day, and 72, on an average, in the night. In the north-east monsoon, on the other hand, the weather was cold and clear. In December, January, and February, 1840, the thermometer often stood as low as 25° in the night, with much ice on the ponds. Very little snow fell in Winter. The position of Chusan is lat. 30 North, and long. 122 East—the climate comparatively mild. Fevers are very prevalent among the natives, all over the island, but especially in the valleys, where the fields are kept long under water. In 1840, however, disease prevailed more extensively than usual—a circumstance tallying with what occurred among the natives of Walcheren during our disastrous expedition in 1809. Some parts of the city are almost pestilential—and malaria exists in almost the whole of the valleys, from the excessive moisture on the surface of the ground. It was no wonder that the laborious duties of our soldiers and sailors—their exposure to the sun by day and the dews by night—their bad provisions—the malaria from the fields, and other causes, should have engendered fevers, dysenteries, and other complaints in abundance.

Mr. Lockhart thinks it very questionable whether Chusan would be more unhealthy than any other place of similar latitude, were it not for the cultivation of the rice by means of stagnant water. By drainage, or even opening the floodgates that dam up the streams, the island would soon become dry and sufficiently healthy.

INTERMITTENT FEVER prevails to a great extent among the inhabitants. Quinine generally checked the disease effectually. The Chinese physicians employ tiger's bones, genseng, &c. with no very great success according to their own confession. OPIUM SMOKING prevails to a considerable extent among the better classes of society in Tinghae. ELEPHANTIASIS is very common, and often commits fearful ravages. OPHTHALMIC diseases, however, are more prevalent in China generally than in any other country of the world. It is difficult to account for these morbid features in the physiognomy of various nations. A friend of our's, when puzzled by such questions, generally remarks that such occult causes can be best explained by a person of the name of FELIX.* Our

* FELIX, qui potuit rerum cognoscere causas.

author thinks that one cause of the great prevalence of ophthalmic diseases is the frequency of inflammation of the eye at the changes of the monsoons.

"2dly. The injurious effects of a practice which is commonly followed by the Chinese barbers of everting the lower lid, and rubbing its inner surface gently with an ivory or bamboo instrument, shaped like a small scoop, which they also pass under the lid and deep into the inner and outer canthi; this they call 'washing the eye,' and the declared intention is the removal of any portion of mucus that may be lodging on its surface. It is a very common habit and performed daily in the barber's shops, where, after the head has been shaved, the man sits composedly as if enjoying exquisite delight, while the barber is thus *operating* on his eyes. If the person's eyes be examined after this process, they will be found to be very red and in a state of considerable irritation, and in process of time chronic conjunctivitis supervenes, and this being considered as the result of the eye not being sufficiently cleansed, the practice is persisted in, and the conjunctiva of the lid becomes covered with granulations. In other cases, the conjunctiva becomes indurated like thin parchment, the tarsal cartilages contract and induce entropium. Other diseases also result in process of time, variously modified according to circumstances; as for instance, exposure to the cold wind inducing an attack of acute inflammation of the organ."

Verily our Chinese *neighbours* have strange notions of the pleasures of cleanliness! We anticipate much benefit, in more ways than one, from such an establishment as the hospital in question. Should a peaceable and commercial intercourse between the Celestial and the British empires be once more effected, the Medical Missionary Society will form a strong cementing link between the two nations—and one that is little likely to be broken by political or mercantile squabbles.

---

TIC DOULOUREUX, OR NEURALGIA FACIALIS, &c. &c. THEIR SEAT, NATURE, AND CAUSE, WITH CASES. By *R. H. Allnatt*, M.D. Octavo, pp. 184. Churchill, 1841.

This is a neat resumé of all that is *known*—we will not say all that has been *written*, on the subject of neuralgic affections. The *seats* of this painful, and somewhat mysterious malady, are nearly as numerous as the various organs and structures of the body. The skin, the muscles, the ligaments, the membranes, the nerves, the viscera, nay, the bones themselves, may and do become the theatres of its agonizing tortures. That dreadful and often fatal disease, angina pectoris, is not improbably the result of neuralgia of the heart itself. M. Piorry has described a curious species of tic, namely, "neuralgie irienne ou ophthalmique," —the pain commencing, as he supposes, in the nerves of the iris, and attacking persons who live in dark apartments—who read much—and whose occupations require the eye to be fixed on minute objects. But, in fact, it would be endless to attempt an enumeration of the Proteian forms of this insidious and cruel malady. Neither need we follow our talented and industrious author in arranging the catalogue of "unsuccessful modes of treatment," from the carbonate of iron down to the solid steel itself, in the shape of the scalpel.

At page 27, our author begins to broach his own doctrine or creed, by joining Bellingeri in the opinion that the trifacial is a vital nerve, from its origin and structure—in short, that it closely resembles the nerves of organic life—and consequently is a nerve of involuntary action. We all know how frequently it is the seat of neuralgia. Sir Charles Bell attributes this to "the influence of the sympathetic nerve," with which it sympathises so closely. Dr. A. next gives a neat COUP-D'ŒIL of the anatomy of the great sympathetic, exhibiting its vast connexions with all the other nerves of the body. In respect to its physiology, the older anatomists were too limited when they ascribed its origin to the fifth and sixth pair of nerves.'

"But subsequent researches have proved this view of its origin to be too limited, and it is now generally admitted that, as expressed by Sir C. Bell, 'it has innumerable origins, and a universal connexion with the other nerves through all the trunk of the body. Many of the viscera to which it is distributed are entirely independent of the will, and have functions to perform too essential to life to be left under the influence of the will. The sympathetic nerve is thus, as it were, a system within itself, having operations to perform of which the mind is not conscious; whilst the extent of its connexion occasions, both in health and disease, sympathetic affections not easily traced.'"

The causes of tic have given rise to great diversity of opinion. We believe that the majority of practical observers are inclined to the opinion that it is attributable to gastric, or gastro-intestinal irritation. So early as 1771, Rahn came to this conclusion, and various physicians and surgeons since that period have adopted his views. We need only select Abernethy and Sir Charles Bell as examples. The following passage will shew Dr. Allnatt's own creed.

"Facial neuralgia and disorder of the chylopoietic viscera almost invariably coexist, and it is often exceedingly difficult, nay, almost impossible, to determine, from the statement of the patient himself, which may have been the primary affection. I have scarcely ever seen a case which was unaccompanied by dyspeptic symptoms. Some of these may have been obscurely developed, but I am firmly of opinion, from observations which I have been enabled to make, that tic douloureux, or facial neuralgia, arises in every instance from an unhealthy condition of the digestive apparatus."

But we must lay aside all speculations, theories, and doctrines, and come to practical facts. From the preceding passages our readers will be prepared for the plan of treatment recommended by Dr. Allnatt.

"Keeping in view the principles I have endeavoured to inculcate in the preceding pages, the indications to be attended to in the treatment of tic douloureux are, to relieve the irritation of the abdominal viscera, and, in cases of long standing, the consequent hyperæmia which may have been induced. For this purpose, I have found the free use of aperients of unfailing efficacy, and I give a decided preference, over all others, to a pill combining a small quantity of croton oil with stomachic aperients.

In plethoric habits, and when the constitution has not materially suffered by protracted agony, the aperient plan should be steadily persevered in and carried to its full extent; that is, the patient may be kept under the influence of purgatives until the pain has subsided.

The diet, which of course must be carefully regulated, should consist of light and nutritious food; all indigestible aliment should be avoided; and irritating spirituous and fermented liquids absolutely prohibited.

Exercise in the open air is particularly desirable, as it tends to the 'equalization of the circulation:' not, however, that exercise which consists in the luxurious rolling of a carriage, but brisk walking on foot until a glow is excited, or, what is still more desirable, horse exercise.

By these means, and these alone, I have succeeded in curing inveterate cases of tic douloureux in the course of six or eight days, which had withstood for months and years every other method of treatment.

But suppose a weak and delicate female, with anæmia, to be the subject of tic douloureux, in whom the periodical functions of the uterus are irregularly performed, or in whom the disorder is complicated with hysteria or other affections connected with an irritable and mobile state of the system—in this case, purgatives must be resorted to with great caution and in very small and divided doses; still they must be used, and alternated, as occasion may require, with ammonia, steel, the vegetable bitters, sedatives, &c. It is in these instances that quina and the sesqui-oxyd of iron produce such marked and decided relief."

The second part or section of the work is occupied with " Other Affections of the Ganglionic System," as hepatalgia—palpitation—sympathetic headache—neuralgia spinalis—hysteria—spasmodic cough—amaurosis—epilepsy, &c. followed by a considerable list of cases, to which we must refer our readers for much research, ingenious reasoning, and practical information.

---

### Medical Reform.

### Sir James Clark's Letter to Sir J. Graham on Medical Reform.

We received this Letter at the eleventh, or rather at the twelfth hour, and had only time to glance hastily over it. It is sensibly and temperately written, though some portions of it are a little obscure. We think the following heads will contain the leading principles and features of the proposed plan of reform.

1. A college, faculty, or ruling body to be formed in each of the three divisions of the empire for examining candidates and granting degrees.
2. The profession to be divided into two grades or classes—Bachelors of Medicine and Surgery, and Doctors of Medicine and Surgery.
3. The education and examination for the Bachelors to be uniform.
4. Both ranks to pass through the examination for Bachelor, whether they mean to attain the higher grade or not. Thus, as medicine and surgery are always more or less combined, so the General Practitioner must pass this ordeal as well as those who are intended for the doctorate.
5. All Bachelors, and of course all General Practitioners, to be eligible for the higher grade, or Doctorate, whenever they submit to the examinations, &c. allotted for that grade.
6. Sir James thinks that a union of the existing Colleges of Physicians and Surgeons, in London, Edinburgh, and Dublin, might constitute the ruling body for the respective capitals.
7. A supreme senate, in connexion with Government, to superintend the concerns of the Colleges and the profession generally. He thinks the present " University of London," with extension and modification, might be easily converted into the supreme senate.
8. The Bachelors, and consequently the body of general practitioners, to have a voice in the election of the councils of the respective Colleges.
9. A court of honour to be vested in the Colleges or Senate, to punish, by striking off the list, such members as disgrace themselves or the profession by misconduct.
10. Sir James thinks it desirable that the *practice* of pharmacy should be divorced from the practice of physic and surgery.

The above are the chief characteristics of the plan proposed, and we need hardly say that they approximate closely to those which we have long advocated in this Journal.

---

### Plan of Medical Reform, &c. &c. By *Rich. Carmichael,* Esq. M.R.I.A, In a Letter to Sir Robert Peel, Bart. Pp. 55. Longman & Co. 1841.

This plan may be comprised in a few lines—the reasons for such plan, and extracts from the report of the parliamentary committee, occupying the 55 pages of the pamphlet.

"I shall not, however, here enlarge upon the reasons that render the improvement and re-organization of the medical profession indispensably necessary. They will be sufficiently obvious by laying before you what the majority of medical reformers require:—

1st—A good preliminary education, such as is required of those who enter into the other learned professions.

2ndly—A good *practical* professional education, to be tested by a scrutinizing *demonstrative* examination.

3rdly—Equality of qualification in each great division of the United Kingdom.

4thly—The union of physic and surgery, at least in education.

5thly—The separation of the practice of pharmacy from the practice of medicine, as far as the interests and usages of society may permit."

Mr. Carmichael states, and truly, that, while almost every person of intellect and candour admits the necessity of medical reform, yet "scarcely even two of the members of the medical profession itself are agreed as to the nature and extent of it." Now if this be the case, and we fear that it is, what hope have we that the head of the government, Sir R. PEEL, and that of the Home Department, Sir James Graham, can disentangle the subject of its infinite perplexities, or reconcile such jarring opinions, when they are badgered on all sides by matters of the highest political interest, affecting the welfare of the state, and the tenure of their own office? Mr. C. may depend upon it that Sir Robert and Sir James Graham will be ear-wigged day and night by interested personages. Reform will be left to partizans, and the consequence will be, we fear, PARTY-LEGISLATION!! Agreeing, as we do, with Mr. Carmichael on all the leading principles of medical reform which he advocates, we see much more difficulty in the execution of those measures than he appears to do. We are quite confident that unless a *high and uniform* standard of education, preliminary and medico-chirurgical, be adopted, the profession will continue to swarm, as it now does, with a multitude of unequally educated aspirants, whose hunger and necessities will compel them to worry and wrangle for a morsel of bread, or a new patient.*

We may here glance at some of the details of the plan sketched out by Mr. Carmichael.

1. The preliminary examination in classics and science should be equal to that which graduates or undergraduates pass through at the Universities.

2. A good *practical* professional education, to be tested by a searching *demonstrative* examination. The number of years passed in hospitals, dissecting-rooms, and lecture-rooms to be not less than five—better to be six or seven. "No portion of this period to be spent in an apothecary's or chemist's shop."

3. The education and tests of examination are to be conducted on the same scale, and in the same manner, in the three kingdoms, so that there may be an equality of qualification throughout Great Britain and Ireland. Upon the question of having two, or only one grade in the profession, Mr. C. observes:—

"This is a question upon which the profession are much divided: some contending that if there is but one grade of practitioners, who must, of course, be required to have a liberal and extensive education, both preliminary and professional, there will not be a sufficient supply of medical men to meet the wants of the nation; others, on the contrary, aver that under the one grade there will be an abundant supply to meet every demand. At present all parts of this vast empire are inundated by hordes of struggling and starving medical men, who have little other occupation than expressing to one another their unavailing regrets that they have spent their time, money, and labour in acquiring the knowledge of a profession which is now of no use to them. The only legitimate

---

* See the disgraceful contest of this kind, as stated in the Medical Gazette for January 7, 1842. Page 605.

way of guarding for the future against this evil is, to raise the qualification, which, by requiring more time, labour, and money than is at present demanded, will tend in a few years to lessen the supply of medical men, and yet render it commensurate with the *real* wants of the people."

"If you admit of two licensed grades of the profession—the one to supply advice to the affluent, the other to afford both advice and medicine to their less fortunate fellow-subjects—you will perpetuate the evil system which now exists, and you will continue to have the nation overrun as hitherto by ignorant or half-educated medical men."

But *even* if all are *permitted and authorized* to charge for their visits, and not to supply medicines, there will be a large number of young and needy practitioners, who will immediately advertise that they will supply medicines and give advice, merely charging for the *former* and giving the latter gratuitously. We apprehend that no law can ever pass *prohibitory* of dispensing medicines by a regularly educated medical man under the new regime—and there will be plenty to take advantage of the non-prohibition. We have long been convinced that nothing but an agreement and consent among the practitioners of each locality, can ever mitigate this evil. We say *mitigation;* for there will be some who will hold out against, or break through the best regulations.

3. Mr. Carmichael advocates, what this Journal has always advocated—the union of physic and surgery, IN EDUCATION—leaving it to the choice of the individual to practise which branch of the profession he pleases afterwards.

"In large cities and communities, some will naturally incline to the practice of physic, while others will prefer that of surgery, and thus individual eminence will naturally be acquired in either one or other of those divisions. But in smaller towns and communities, both branches must be practised by the same persons; and for this indispensable necessity they will, under the proposed reform, be amply qualified."

4. The last question is—"the separation of the practice of pharmacy from that of medicine—*as far as the interests and usages of society may permit.*" Mr. C. considers this the most difficult question of all, and without this reform, all others "would be a mere bagatelle."

"No one can doubt that the same individual who is busied all day in visiting and prescribing for patients cannot superintend as he ought the preparation and compounding of medicine: hence the numerous fatal mistakes, not one in a hundred of which ever meets the public eye, notwithstanding the numerous instances so constantly detailed in the diurnal press. No one can doubt that, if an apothecary or general practitioner is not paid for his visits or attendance, he must prescribe medicine in such mode, and in such quantities, as will enable him to live."

It unfortunately happens that nine-tenths of those who are loudest in their clamours for medical reform, are inimical to this separation of physic from pharmacy, without which separation Mr. C. considers everything else as bagatelles. Still he thinks that although the unhappy junction of physic and pharmacy cannot be abolished by law in this country, where it has taken such deep root, yet that it may be modified, "so as to preserve all its utility to the public, and, at the same time, deprive it of its mischievous tendencies." This happy medium —this "juste millieu," is comprised in the following sentence.

"This may be done simply by a legislative enactment that all general practitioners *shall be entitled to charge for attendance, but not for medicine;* and it may also be provided, that any general practitioner who shall be convicted of sending out medicine from his shop or laboratory that has not been compounded either by himself or by some *licensed* apothecary, shall be liable to the punishment of fine or imprisonment."

We thought Mr. Carmichael knew human nature better, and the discontent of sick folks better than is evinced in the foregoing passage. If general practitioners charged only for their *attendance*, and threw in their medicine *gratis*, the patient would instantly conclude (for the ingratitude and suspicions of this class are boundless) that the doctor would be extremely liberal of his *visits*, but wonderfully chary of sending a particle of *medicine* that was fit for a dog to swallow! We have been longer conversant with the sick-room than Mr. Carmichael, and we *know* that this is the very sentiment that would pervade half the community of invalids. No. It is better to separate the two branches entirely, or to charge for "medicine and attendance," from 3s. 6d. to 7 shillings per diem, according to the severity of the case, or the distance traversed.

It is obvious that the reform proposed by Mr. Carmichael would at once do away with the whole system of apprenticeship; and here again a great majority of the present race of general practitioners will strenuously oppose the plan. In short, whichever way we turn, we see nothing but difficulties—nothing but clashing interests—nothing but contrariety of sentiment on almost every point of medical reform! We have seen the fate of the general Reform Bill, which is now almost as much abhorred by the Whigs and Radicals, as by the Tories and Conservatives! We confess that we anticipate little but abortive measures in the regeneration of our own profession—BY LAW. If reform began at home, and men treated their brethren like brother-practitioners, with liberality and kindness, medicine would rise to a far more dignified station that it has as yet attained. Parliamentary enactments will do little good till after the moral reform has been effected.

The formation of a SENATE, however, which may control the Colleges, and regulate the profession generally, is calculated, we think, to effect much good. This is the only effective measure which we expect from Sir J. Graham's new Bill.

---

A PRACTICAL TREATISE ON THE CURE OF DISEASES BY WATER. By *James Wilson*, M.D. Physician to Prince Nassau, &c. Churchill, 1842.

Pindar little expected that his dogma—"ariston men udor"—would be the creed of the nineteenth century! The water companies are in a panic—not from any fear of losing their customers—but from the dread of a *run* upon their reservoirs by the tea-totallers on one side, and the hydropaths on the other! Meetings, we understand have been held among the water companies to consider the means of warding off, or rather of meeting the impending storm. One of the projects, recommended by the THAMES Company, is to leave off filtering or depurating the water, and to supply it just as it is pumped up from the river at high tide, when the stream has completely washed out the common-sewers, and carried their contents up to the pump-engine at Chelsea. They think, and with some reason, that if this plan does not sicken the disciples of Priessnitz, nothing else will!—The New-river and other companies think of infusing as much ipecacuanha or tartrate of antimony into their sources as will nauseate the hydropaths, without sickening their *temperate* customers.

Meantime we think we can allay the apprehensions of the water companies—and also of the Apothecaries' Company, by assuring them that the wet sheets of Graefenberg will never become popular in England—nor will the ingurgitation of Thames water long supersede that of "Truman, Hanbury, and Co." In fact, we have little doubt but that Priessnitz himself, after draining a pot of the real "heavy wet," would abandon his "kalt wasser" and wet sheets for ever.

It will be quite unnecessary for us to go into argument with Dr. Wilson. The following specimen of the reasoning, or rather declamation, which fills nine-tenths of the volume, will give the reader some idea of its contents.

"How often have I observed the *undertaker's house* placed between a gin palace and a druggists's shop, and heard at the same time the curse and drunken hiccup,—the undertaker's hammer,—and the pestle and mortar of the druggist, —blending into strange unison, and producing a combined sound, which, when modulated and softened down by distance, came on the ear like an unearthly wail of 'Woe! woe!' "

In answer to the remark that has been made that there is nothing *new* in Priessnitz's cold water cure, Dr. W. replies, "is there nothing *new* in curing inflammation of the lungs or apoplexy with cold water?" We say there is certainly something new in such a cure—but then M. Priessnitz must first prove to us that there is also something *true* in the averment.

Why did not Dr. Wilson ask us the following question. "Is it nothing *new*, when M. Priessnitz cures Etna and Vesuvius of fire-spitting, by pouring down their throats the Mediterranean Sea?" We answer that there is just as much of the *new and the true* in this cure as in the other. It is acknowledged, even by Mr. Claridge, that Priessnitz is so ignorant as not to know in which side of the body the liver is located. If so, how could such a man diagnose pneumonia or pleuritis from neuralgia or rheumatism of the intercostal muscles? We have known men, *melioris notæ*, make such mistakes, but as for the Silesian peasant, it is absurd to suppose that he could tell carditis from colic.

The cases of acute inflammation said to be cured by Priessnitz are, in all probability, deceptions—we do not say wilful ones. In some constitutions, such is the power of the vis vitæ, that nature will cure diseases, not only without proper remedies, but in despite of improper ones. If acute diseases ever get well in wet sheets at Graefenberg, it is owing to the strength of the constitution, and not to the virtue of cold water, either inwardly or outwardly applied.

Dr. Wilson avers that he *has seen* pneumonia, "where nearly the whole substance of the lungs was inflamed, as shewn by the stethoscope, cured in 38 hours." Now we ask him, did this patient climb the mountains and repair to Graefenberg, with nearly the whole of the lungs in a state of acute inflammation? If the pneumonia was contracted during the water-cure at Graefenberg, what says Dr. Wilson to the remedy which could cure the inflammation but not prevent it? But the climax of absurdity is not yet reached. Dr. W. asserts that he has seen APOPLEXY cured in a few hours by Priessnitz!!! Now did the patient climb up to Graefenberg in a state of apoplexy? Or did he become apoplectic while there, under cure for some other complaint?

We need go no farther. Dr. Wilson has no doubt that "establishments for the treatment of diseases by water will be formed in *every part* of England in a very short time." Gently good Doctor! Remember the fate of St. John Long. There is such a personage, in each county, as a coroner; and, as *water-curing* in England will be little else than *water-killing*, we advise the hydropaths to have the fear of God and of Mr. Wakley before their eyes!

---

THE ANATOMY OF THE URINARY BLADDER AND PERINÆUM OF THE MALE. Illustrated by Engravings. With Physiological, Pathological, and Surgical Observations. By *Alexander Monro*, M.D. F.R.S.E. &c. 8vo. pp. 90. Edinburgh: Maclachlan, and Co. 1842.

DR. MONRO's work is likely to prove very useful to students, whether of younger or of later growth. In it we see displayed the mixture of anatomical description with practical remarks, which is always so instructive.

For example, the ischio-rectal fossa is described, with its walls composed of the ischio-rectal layer of fascia covering the levator ani on the inner side, and the fascia investing the lateral surface of the obturator muscle on the outer side.

These layers of fascia run into one another above. On this, Dr. Monro observes, that the junction of these two layers superiorly seems to account for the internal orifices of fistulæ, being situated generally at such a short distance above the anus, because, owing to the resistance offered by these fasciæ superiorly, matter is prevented from burrowing in that direction, and the abscess opens either into the lower part of the rectum, or at a short distance from the anus, or near the tuber-ischii.

He gives a very good account of the superficial perineal fascia, and concludes: —In reviewing the description of the superficial fascia, it is of the highest importance to bear in mind its various attachments, and to reflect, how these operate in cases where urine has been infiltrated, either from rupture or ulceration of the membranous part of the urethra. In such cases, the effused fluid, instead of passing down the thighs or towards the ischio-rectal region, never passes lower than on a level with the apices of the tuberosities of the ischia, and then proceeds upwards to the lower part of the abdomen, infiltrating the scrotum and penis. Now on looking over the description of the fascia, the causes are at once obvious; the superficial fascia is dense and resisting, its attachments laterally to the rami of the pubis and ischia effectually prevent fluid passing in that direction towards the thighs, whilst its reflection posteriorly and its union there with the deep fascia prevent it passing towards the anal region; but its continuity anteriorly with the loose cellular fascia of the scrotum, readily allows the passage of fluid in that direction; and this shews the absolute necessity of having recourse to early and free incisions through this fascia in cases of infiltration of urine, as affording the only chance of success, by giving free vent to the confined acrid fluid, and the sloughing cellular tissue.

Speaking of the *Bladder*, Dr. Monro observes:—By disease, the form of the bladder is materially altered; thus, from the irritation of a stone, it is sometimes contracted, and its coats are at the same time thickened, that it cannot be made to rise above the ossa pubis, so that the patient has very frequent calls to pass urine; or, it is contracted upon a stone, in the middle, like a sand-glass, or divided, in its under part, into two lateral portions. Hence, before attempting the operation of lithotomy, it is of the greatest moment to ascertain whether the bladder will rise above the ossa pubis. Thus we learn whether the coats of the bladder be in their sound and distensible state, or not.

He seems to be of opinion that division of the *pelvic fascia* is not so fatal, in the operation of lithotomy, as has been supposed. "Considering," he says, "that stones weighing 13 or 14 ounces have been extracted, and with no other bad consequence than incontinence of urine, as in a case operated upon by Mr. Alexander Wood, and in another instance recorded by Klein, who extracted a stone which weighed 12 ounces and 30 grains, and whose patient recovered,—and in other cases recorded;* it seems to me probable, that the division of the pelvic fascia does not invariably prove fatal. This supposition seems the more probable, from the following statement by Mr. Martineau of Norwich, one of the most successful lithotomists, who has observed, 'Should the stone be large, or there be any difficulty in the extraction, rather than use much force, while the forceps have a firm hold of the stone, I give the handles to an assistant, who is to draw them upwards and outwards, while the part forming the stricture is cut. I have often repeated this enlargement.'"

It is right to observe, however, that this statement of Mr. Martineau's has given rise to some astonishment on the part of those who saw him operate.

*Sounding for Stone.*—If at at last, says Dr. Monro, after describing the process, we rub upon some hard substance, we are not at once to take it for certain

* Vide Med. Chir. Trans. of London, vol. xi. p. 54.

that there is a stone in the bladder, on account of which lithotomy is necessary; for a constriction and induration of the neck of the bladder has sometimes deceived surgeons; and they may still more readily, and in fact have often been deceived by small particles of sand or gravel sticking about the neck of the bladder, or within the urethra, or lodged in small cysts in the sides of the urethra, or in the prostate gland.

"Before dismissing this subject of sounding for a stone, I will venture to allege, that surgeons have, very generally, committed a mistake, which has been the occasion of much controversy among them, in supposing, that in sounding, or in introducing a catheter, success depends much on the posture of the patient, instead of which, as the bowels within the pelvis, through which the sound passes, are all fixed in their places, their relative situations must be the same in all postures, and, in fact, we may observe that different surgeons not only attempt to sound their patients, in the different postures of standing, sitting, or lying, but succeed equally well in all of them. Changing the posture of the patient can serve only to change the posture of the stone."

But surely this is just the thing to be desired. The stone is apt to fall down behind the orifice of the urethra, and here it is difficult to reach it. By raising the pelvis, and injecting water into the bladder, the stone is brought to gravitate to the posterior part of the bas fond of the bladder where the sound can get at it.

*Hæmorrhage from Lithotomy.*—Dr. Monro quotes from some very valuable remarks by Mr. Spence, assistant demonstrator in the University, who writes from the examination of seventy-three subjects.

*The Internal Pudic Artery.*—After enumerating several cases in which the pudic artery had been wounded by celebrated lithotomists, Mr. Spence continues. "It is, however, worthy of remark, that in all the cases mentioned, with the exception of that by Mr. Crosse, who does not give the particulars of the operation, the instruments used to divide the prostate were either the cutting gorget, Blizard's beaked knife, or the lithotome caché; the method of using which, and the danger arising from it, I have already explained. And after mature consideration of the surgical relations of the vessel, and of the cases in which it has actually been wounded, I cannot help thinking that, if the operation be performed with the knife, the staff held steady by an assistant from first to last, and the prostate divided obliquely downward and outwards, a wound of the pudic artery will be a rare occurrence indeed."

*Irregularity of the Pudic Artery.*—In one subject Mr. Spence found a large vessel arising from the internal iliac in common with the obturator; it then passed along the side of the bladder, and over the upper surface of the prostate; on arriving near the pudic arch, it pierced the fascia immediately external to the left anterior true ligament of the bladder, and divided into three branches; one entered the spongy part of the urethra, about an inch anterior to the bulb; the other two branches were distributed, one to the dorsum, the other to the crus penis. If such an anomaly as that described in either of the two first-mentioned cases existed on the left side of a person who was to undergo the lateral operation, the artery must inevitably be wounded either in opening the urethra or on dividing the prostate. But, in this particular instance, the vessel would have been in no danger, for it lay completely above the line of incision, and on the upper surface of the prostate.

*Irregularity of the Artery of the Bulb.*—"In a subject which I dissected in 1837, the artery of the bulb arose from the pudic as usual, but then passed almost directly backwards to near the anus, whence it again curved upwards to gain the bulb. I have also seen two cases similar to that described by Mr. STANLEY, in which the vessel came off from the pudic posterior to its usual origin, ran immediately above the inferior margin of the triangular ligament, and then passed upwards to the bulb. It is evident that in such cases, and also where the vessel comes off from the irregular pudic trunk and runs along the membranous part of the urethra, as in the case mentioned by Dr. MONRO, of which I have already spoken, this artery must be divided in the lateral operation; and the existence of these anomalies sufficiently disproves Mr. LISTON'S sweeping assertion, that the artery of the bulb runs no risk, whatever be its course, if the incisions be made low in the perinæum."

*The Prostatic Artery* arises sometimes as a distinct branch from the internal iliac, but more generally in common with the vesical, or from the internal pudic, in the first part of its course, before it leaves the pelvis. In the great majority of cases the vessel passes along the lateral and inferior surface of the bladder towards its neck, then pierces the ileo-vesical fascia and gains the side of the prostate, on which it divides into numerous twigs, which supply that gland and the neighbouring surface of the rectum. In such a distribution of the artery it is not likely to furnish much blood if divided; but in several instances Mr. Spence has seen the prostatic artery gain the perineal surface of the prostate without dividing into minute branches; and in eight of these cases the vessel was fully as large as the artery of the bulb, and would have bled profusely if divided, as it must inevitably have been in the lateral operation of lithotomy.

*Inferior Hæmorrhoidal Artery.*—"This vessel leaves the pudic opposite the tuber ischii, and is of a considerable size at its origin; but shortly after piercing the fascia which binds down the pudic, it divides into several branches, which pass across the ischio-rectal space, and again sub-divide as they approach the levator ani; some pass into and through that muscle to the lower part of the bowel—others are distributed about the anus. In a few cases, I have seen the vessel pass almost across the ischio-rectal space without dividing into branches; and as it must always be cut in lithotomy, I should think that, in such a case, it would bleed profusely, both on account of its own size, and its proximity to the pudic trunk. I believe, however, that in most cases the artery could be readily enough secured, unless it be cut so close to its origin as to retract within the fascia, or its coats so diseased as not to hold a ligature, a state in which I have frequently found the hæmorrhoidals in old people; and Mr. LISTON mentions a remarkable case of fatal hæmorrhage from this state of the diseased arteries."

*The Prostatic Veins.*—When we consider that these communicate freely with the superior prostatic plexus, and inferiorly with the middle hæmorrhoidal veins, and remember the want of valves in these vessels, and their dilated form in old persons, it must be allowed, that, in them, at least, this plexus constitutes a formidable source of hæmorrhage after lithotomy. And of late years it has been noticed as such by Baron DUPUYTREN, Dr. MONRO, MM. VELPEAU and ROBERT, the last of whom has recorded two cases of hæmorrhage from this source.

Mr. Spence winds up by saying,—"I would, however, observe before concluding, that the vessels which I consider the most likely to give rise to such an accident, are the artery of the bulb and the artery of the prostate when large. For I have found, during my investigations, three cases of irregularity in the artery of the bulb, and eight of the enlarged prostatic artery; so that, in eleven cases out of the seventy-three subjects dissected by me, these vessels must inevitably have been wounded, owing to their position. I also think that these facts are sufficient to prove *that hæmorrhage may sometimes occur, without any fault on the part of the operator.*"

